# Risk factors of local control in adrenal metastases treated by stereotactic body radiation therapy - a systematic review and meta-analysis

**DOI:** 10.3389/fonc.2023.1193574

**Published:** 2023-11-17

**Authors:** Xuehong Liao, Kazushi Kishi, Kaixin Du, Ritsuko Komaki, Junetsu Mizoe, Gosuke Aikawa, Wei Zheng, Chao Pan

**Affiliations:** ^1^ Department of Pathology, Zhongshan Hospital, Xiamen University, Xiamen, China; ^2^ Department of Pathology, School of Medicine, Sapporo Medical University, Sapporo, Japan; ^3^ Department of Radiation Oncology, National Disaster Medical Center, National Hospital Organization (NHO), Incorporated Administrative Agency 3256 Tachitawa City, Tokyo, Japan; ^4^ Department of Radiation Oncology, Xiamen Humanity Hospital Fujian Medical University, Xiamen, China; ^5^ Department of Radiation Oncology, Faculty of Medicine, Hokkaido University, Sapporo, Hokkaido, Japan; ^6^ Department of Radiation Oncology, Emeritus of The University of Texas M.D. Anderson Cancer Center, Baylor College of Medicine, Houston, TX, United States; ^7^ Department of Sapporo High Functioning Radiotherapy Center, Hokkaido Ohno Memorial Hospital, Sapporo, Japan

**Keywords:** adrenal metastases, SBRT (stereotactic body radiation therapy), fractionation dose, tracking, oligometastases

## Abstract

**Purpose:**

This study is aimed to explore risk factors affect the therapy outcomes of adrenal metastases (AM) for stereotactic body radiation therapy (SBRT) and guide clinical dose selection.

**Methods and materials:**

PubMed, Embase and Web of Science were searched in September 22, 2022 in accordance with Preferred Reporting Items for Systematic Reviews and Meta-Analyses guidelines (PRISMA). Subgroup analysis and meta-regression were used to search for sources of heterogeneity and identify risky outcomes factors. Publication bias test and sensitivity analysis were also conducted.

**Results:**

Thirty-three studies with full text from 2009 to 2022 about AM with SBRT on 1483 patients were included. Pooled 1- and 2-year local control (LC) and overall survival(OS) were 81.7% (95% confidence interval [CI], 75.6%-86.5%), 62.8% (95% CI, 53.8%-71.8%), 67.4% (95%CI, 61.8%-73.1%) and 46.5% (95%CI, 40.4%-52.6%), respectively. Biological effective dose (BED, *α/β*=10Gy) and dose per fraction affected 1-year LC (Qm=23.89, 15.10; *P*<0.0001, 0.0001). In the range of 60-80Gy (BED_10_), the group of dose per fraction ≥ 9Gy achieved the excellent 1-year LC (< 9Gy: ≥ 9Gy =78%, 91%; χ*^2^ = *10.16, *P* = 0.001). Tracking technology significantly affected 1- and 2-year OS (Qm = 5.73, 8.75; *P* = 0.017, 0.003) and high tracking adoption group showed excellent 1- and 2- year OS (78.7% [95%CI, 68.6%- 88.9%]; and 62.9% [95%CI, 53.1%-72.7%]).

**Conclusion:**

Increasing the dose per fraction appropriately may help control locally AM lesious. Tracking technology might contribute to improve survival of advanced patients with AM. But these results need prospective studies to verify them.

## Introduction

The adrenal glands are a common site of metastasis., accounting for up to 38% of metastastatic cancers (1), with melanomas (50%), lung and breast cancers (30-40%), and renal and gastrointestinal tumors (10-20%) (2) being commonly affected due to the adrenal glands’ rich blood supply (3). Advanced imaging and close follow-up programs for cancer patients have led to a rise in their detection, typically at the oligometastatic stage. Targeted local therapies are gaining popularity as ablative therapy may improve outcomes in patients with limited systemic disease burden (1, 2). A paired analysis of 62 patients with isolated AM found that SBRT and laparoscopic adrenalectomy had similar outcomes and survival rates (4), but complications occurred in 37.9% of patients, including ileus or gastroparesis, wound problems, pneumonia, and heart arrhythmia (5). Stereotactic body radiation therapy (SBRT) enables conformal delivery of ablative radiation doses using single or a small number of fractions, resulting in a more effective radio biologic dose (6). SBRT has become an important treatment option for adrenal metastases, with its low toxicity and ability to maintain adrenal function.

However, there are currently no specific clinical guidelines established for SBRT in AM. This has resulted in considerable diversity in prescription doses and fractionation plans in clinical settings (7), with notable fluctuations in 1-year and 2-year LC rates, spanning from 44% to 100% and 27% to 100%, respectively (8) Consequently, it is critical to investigate risk factors and develop reliable models to guide clinical dose selection.

## Methods and materials

We conducted a systematic search for relevant studies in PubMed, Embase, and Web of Science, including those published up to July 2022 and updating the search until September 22, 2022. Thirty-three studies published between 2009 and 2022 were included, covering 1483 patients with 1660 lesions (3, 7, 9–38). The screening flow diagram was shown in **Supplementary Figure A** and clinicopathologic characteristics in **Supplementary Table A**. The search query used was “stereotactic OR radiosurgery OR sbrt OR sabr OR knife) AND (adrenal/exp OR adrenal) AND (metastasis/exp OR metastasis OR metastases/exp OR metastases OR metastatic)” (39). We excluded studies that did not report treatment outcomes or toxicity data specific to SBRT for AM, studies that did not grade toxicities according to Common Terminology Criteria for Adverse Events (CTCAE) criteria or define radiological responses according to the Response Evaluation Criteria in Solid Tumors criteria (RECIST) criteria, studies without BED_10_ information, redundant data or those reporting on fewer than 5 patients, or non-English reports were excluded. We followed PRISMA guidelines. Survival outcomes were calculated per patient, while the LC rate was calculated per metastatic lesion. Some outcome values were obtained from local control probability curves using *Engauge Digitizer*. SBRT was defined as the delivery of higher fractional doses of radiation than conventional fractionation (>1.8-2.5Gy) in a relatively small number of fractions (39). Local control was defined as the absence of progression at the treatment site. Oligometastatic disease defined as limited metastatic disease burden with 5 or fewer lesions in 2 or fewer organ sites. Synchronous metastases were defined as those present at the time of first diagnosis or appearing within 6 months. Since some criteria differed between studies, we performed simple calculations, such as calibrating technology adoption rates based on patients rather than lesions in some studies (7, 32). Biological effective doses were calculated by following quation:


BED=nd[1+d/(a/β)]

where *d* is the single radiation dose, *n* is the number of fractions, and we picked up *α/β* =10Gy.

A pooled analysis based on maximum likelihood estimation was used to determine weighted study level rates of 1,2-year LC and 1,2-year OS, and subgroup analysis and meta-regression were conducted to explore possible sources of heterogeneity among study results. Qm was used to assess the degree of heterogeneity (variation) among the effect sizes of individual studies. Significant risk factors identified through meta-regression testing also need to undergo collinearity detection. We reported the total number of adverse events rather than estimating a pooled statistic because the reported rates of grade 3+ toxicity were uniformly low and frequently zero.

### Statistical analysis

A pooled analysis based on maximum likelihood estimation was used to determine weighted study level rates of 1,2-year LC and 1,2-year OS. The metaprop function in the meta package (version 5.5-0) in R was used to perform a meta-analysis of binomial proportions (version 4.2.1). Subgroup analysis and meta-regression were respectively conducted using meta and metaregression function in the meta package. Variable correlation examination was performed by using *scatterplotMatrix* function in the car package (version3.1.0). Qm was used to assess the degree of heterogeneity (variation) among the effect sizes of individual studies. Cases with missing covariates were automatically excluded from regression analyses. The difference in BED10 between different groups was compared using an independent samples t-test by SPSS (version 22.0).

## Results

Out of 915 records, we included 33 studies published between 2009 and 2022 that reported outcomes for 1483 patients with 1660 lesions of AM. The median follow-up was 13.4 months, ranging from 0 to 124 months, and the median OS was 19 months, ranging from 0.4 to 171 months. The median total dose was 36Gy(10-70Gy) and the median number of fraction was 4.5(1-18). The median dose per fraction was 7.5Gy, ranging from 2 to 30Gy, and the median BED10 was 71.4Gy, ranging from 20 to 180Gy. Among the patients, 61% had were primary lung cancer patients, and 91% were oligometastatic or oligoprogressive patients. Ninety-four percentage of the studies included data on the method of diagnosis, and only three studies reported biopsy tissue confirmation along with fiducial placement. Out of 1483 patients, only 19 (1.3%) patients reported grade3+ toxicity response, and out of 1401 patients, 22 patients (1.6%) experienced adrenal insufficiency. For more detailed information, please refer to **Table A in the Supplement Tables**. The pooled 1- and 2- year LC were 82% (95% CI, 76%-87%) and 63% (95% CI, 54%-72%), respectively, and the pooled 1- and 2- year OS were 67% (95%CI, 62%-73%) and 47% (95%CI, 40%-53%), respectively. The forest plots, funnel plots and sensitivity analysis were shown in **Figures B–D in the Supplement Figures**.

### BED_10_ and dose per fraction were significant influence factors of LC

**Figure 1:**
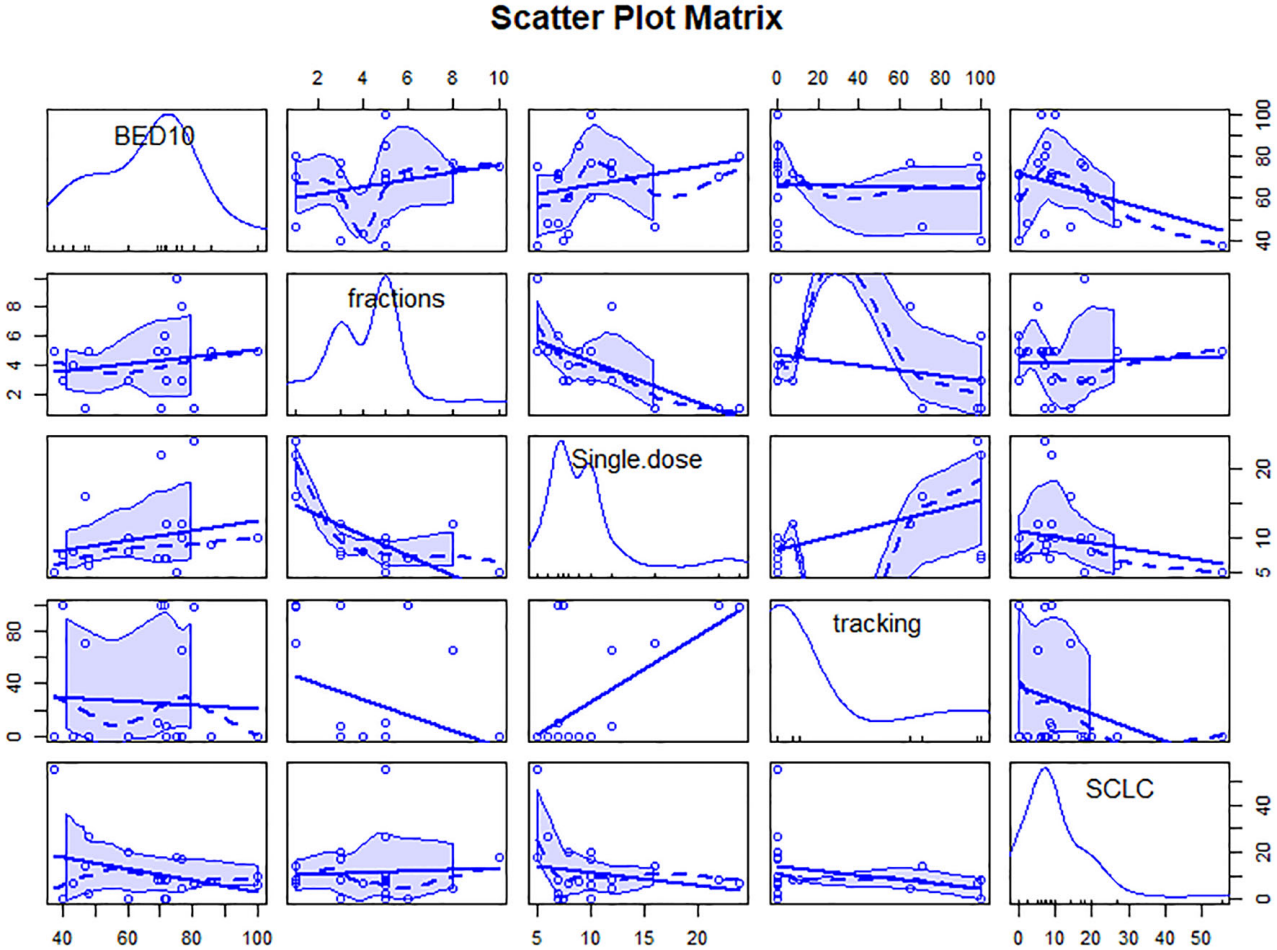
Scatter plot matrix of correlation between variables. The figure contains linear and smooth fitted curves, and corresponding marginal distributions (kernel density maps and axonal whisker maps). single.dose=dose per fraction.

We analyzed the variables about technique, prescription dose and treatment method, or fractionation function to identify sources of heterogeneity for pooled 1- or 2-year LC. As shown in **Table 1**, BED_10_, dose per fraction and SCLC were significant influence factors of 1-year LC (Qm = 23.89, 15.10, 4.37; *P<*0.001,< 0.001, 0.037). Only BED_10_ play a crucial part for 2-year LC (Qm = 7.72; *P* = 0.006). We analyzed the correlation and showed there were moderate negative relation between SCLC and BED_10_ (The coefficient= -0.34, **Figure 1**), which might suggest the effect on 1-year LC by SCLC related to low BED_10._ On the other sides, BED_10_ have a low correlation with dose per fraction (The coefficient =0.24, **Figure 1**). In conclusion, total dose and dose per fraction should be most important factors for LC.

**Table 1:** Analysis of clinicopathological factors affecting the outcome by meta regression.

Variable	Moderators	d.f.	Qm	p-value	Variable	Moderators	d.f.	Qm	p-value
1-year local control	Age	1(27)	0.01	0.926	1-year overall survival	Age	1(28)	0.85	0.356
	Male	1(25)	0.68	0.409		Male	1(26)	0.50	0.479
	Right adrenal	1(21)	0.74	0.391		Right adrenal	1(22)	0.00	0.969
	Bilateral	1(27)	2.01	0.156		Bilateral	1(28)	0.05	0.823
	Concurrent therapy	1(8)	2.53	0.112		Concurrent therapy	1(11)	0.01	0.905
	SCLC	1(20)	4.37	0.037		SCLC	1(20)	2.40	0.121
	Melanoma	1(24)	0.07	0.791		Melanoma	1(24)	1.31	0.253
	Solitary	1(16)	2.94	0.087		Solitary	1(17)	0.42	0.519
	Synchronous	1(15)	1.52	0.218		Synchronous	1(15)	0.21	0.650
	Prescribed dose	1(26)	1.20	0.273		Prescribed dose	1(27)	0.11	0.741
	Fractions	1(25)	1.41	0.236		Fractions	1(26)	1.80	0.180
	Dose per fraction	1(26)	15.10	<.001		Dose per fraction	1(27)	3.10	0.078
	BED_10_	1(27)	23.89	<.001		BED_10_	1(28)	1.95	0.163
	PTV	1(18)	2.89	0.089		PTV	1(18)	0.15	0.703
	GTV	1(25)	0.72	0.396		GTV	1(26)	1.98	0.160
	Tumor size	1(7)	0.03	0.861		Tumor size	1(7)	0.52	0.473
	Tracking technology	1(25)	3.31	0.069		Tracking technology	1(26)	5.73	0.017
2-year local control	Age	1(24)	0.17	0.684	2-year overall survival	Age	1(27)	2.40	0.121
	Male	1(22)	0.48	0.490		Male	1(25)	0.02	0.895
	Right adrenal	1(18)	0.51	0.473		Right adrenal	1(21)	0.39	0.534
	Bilateral	1(24)	1.02	0.313		Bilateral	1(27)	1.33	0.249
	Concurrent therapy	1(8)	0.09	0.761		Concurrent therapy	1(11)	1.65	0.199
	SCLC	1(17)	0.03	0.871		SCLC	1(19)	10.04	0.002
	Melanoma	1(21)	1.03	0.310		Melanoma	1(23)	0.03	0.863
	Solitary	1(15)	1.85	0.174		Solitary	1(17)	0.68	0.409
	Synchronous	1(13)	0.32	0.570		Synchronous	1(15)	1.69	0.194
	Prescribed dose	1(23)	0.82	0.365		Prescribed dose	1(26)	0.10	0.755
	Fractions	1(22)	0.23	0.629		Fractions	1(25)	3.21	0.073
	Dose per fraction	1(23)	1.35	0.245		Dose per fraction	1(26)	6.60	0.010
	BED_10_	1(24)	7.72	0.006		BED_10_	1(27)	2.82	0.093
	PTV	1(16)	0.47	0.491		PTV	1(17)	4.87	0.027
	GTV	1(24)	0.13	0.718		GTV	1(26)	8.20	0.004
	Tumor size	1(6)	1.42	0.233		Tumor size	1(7)	1.27	0.261
	Tracking technology	1(22)	0.34	0.559		Tracking technology	1(25)	8.75	0.003

SCLC, small cell lung cancer; PTV, planning target volume; GTV, gross tumor volume. The red shading mean that significantly difference (p <0.05).

**Figure 2:**
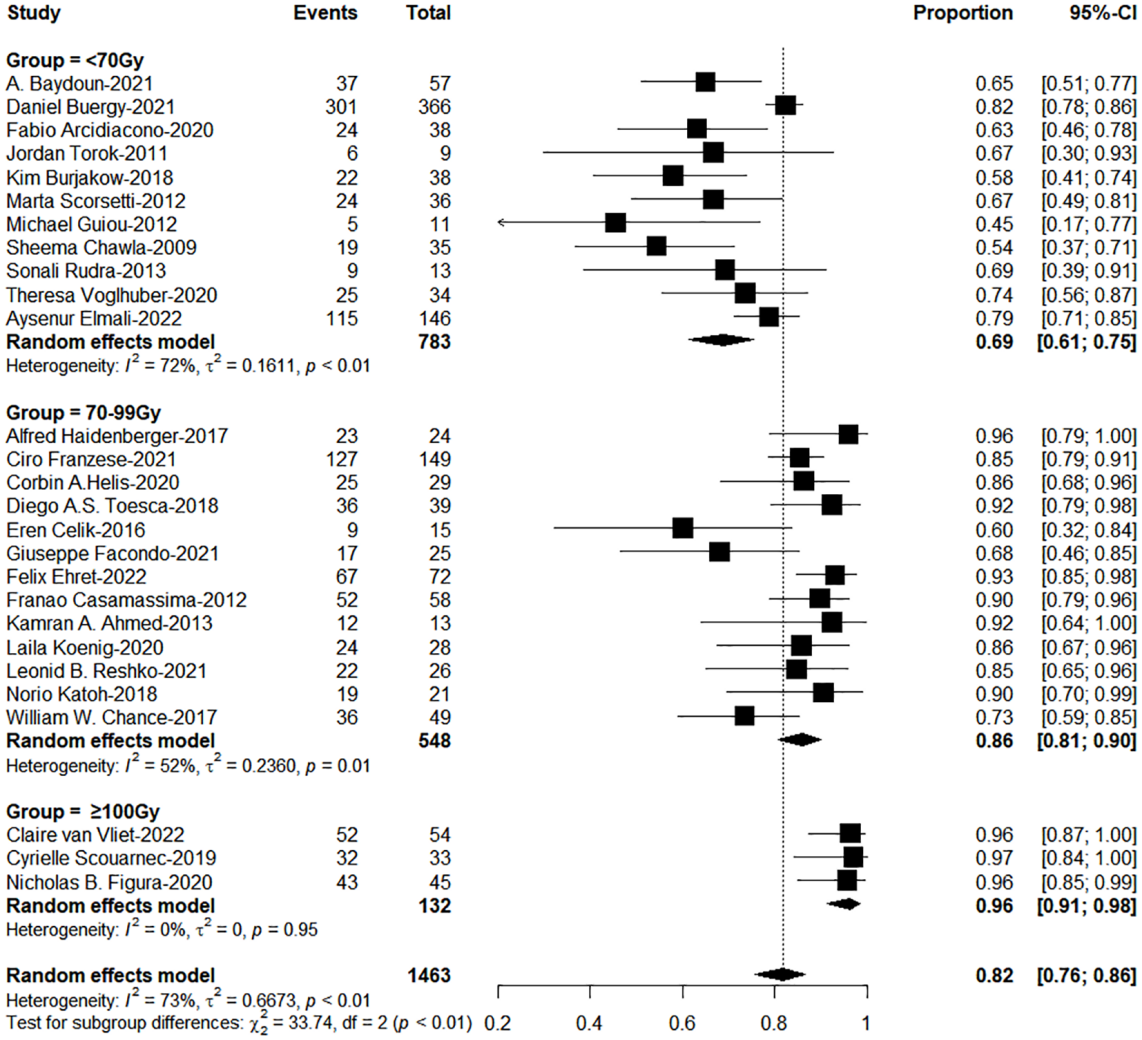
The forest plot of subgroup analysis for one-year local control by biological effective dose (BED, α/β=10Gy).

**Figure 3:**
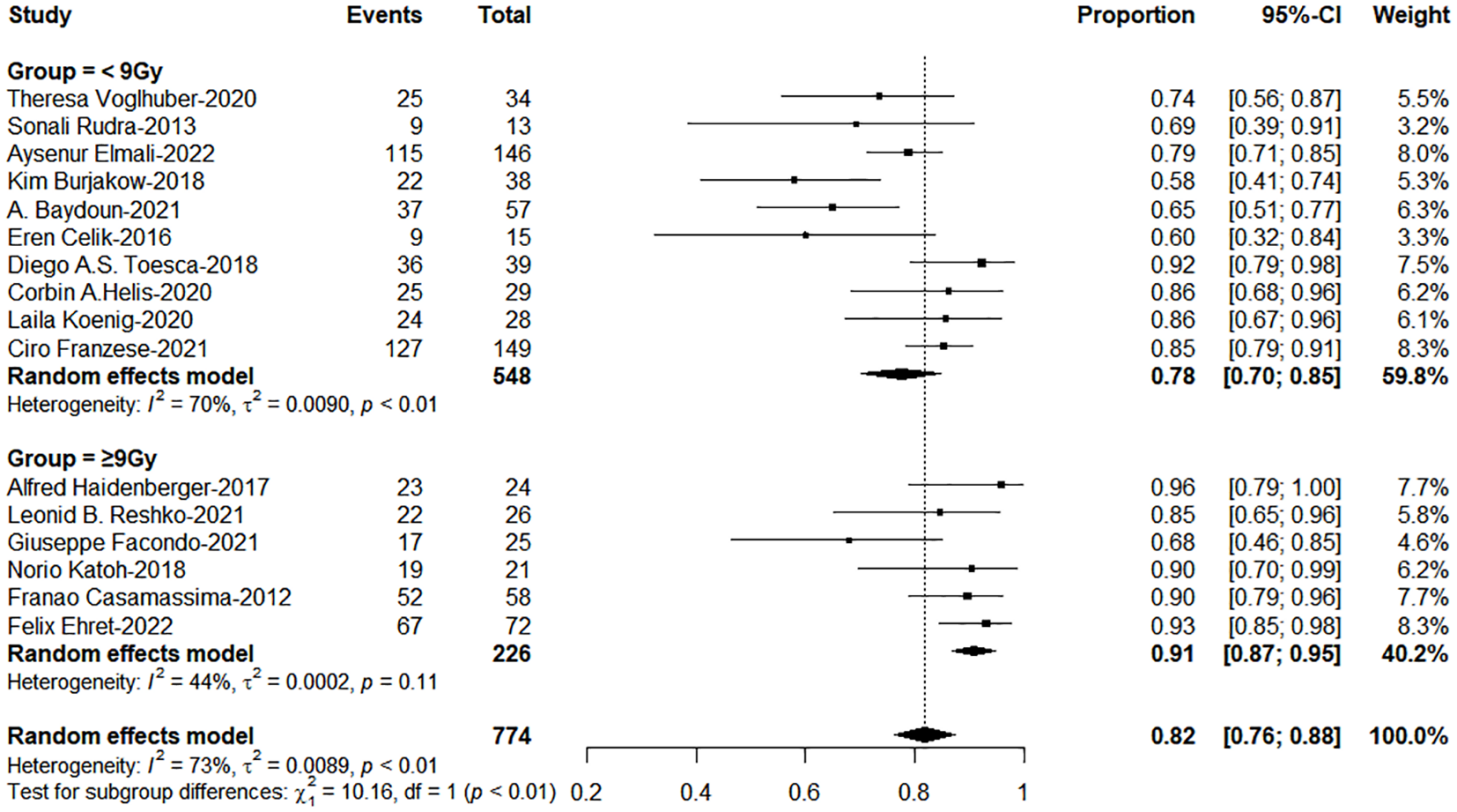
The forest plot of subgroup analysis for 1-year local control by dose per fraction with the range of Biological effective dose (α/β=10)(60-80Gy).

**Figure 4:**
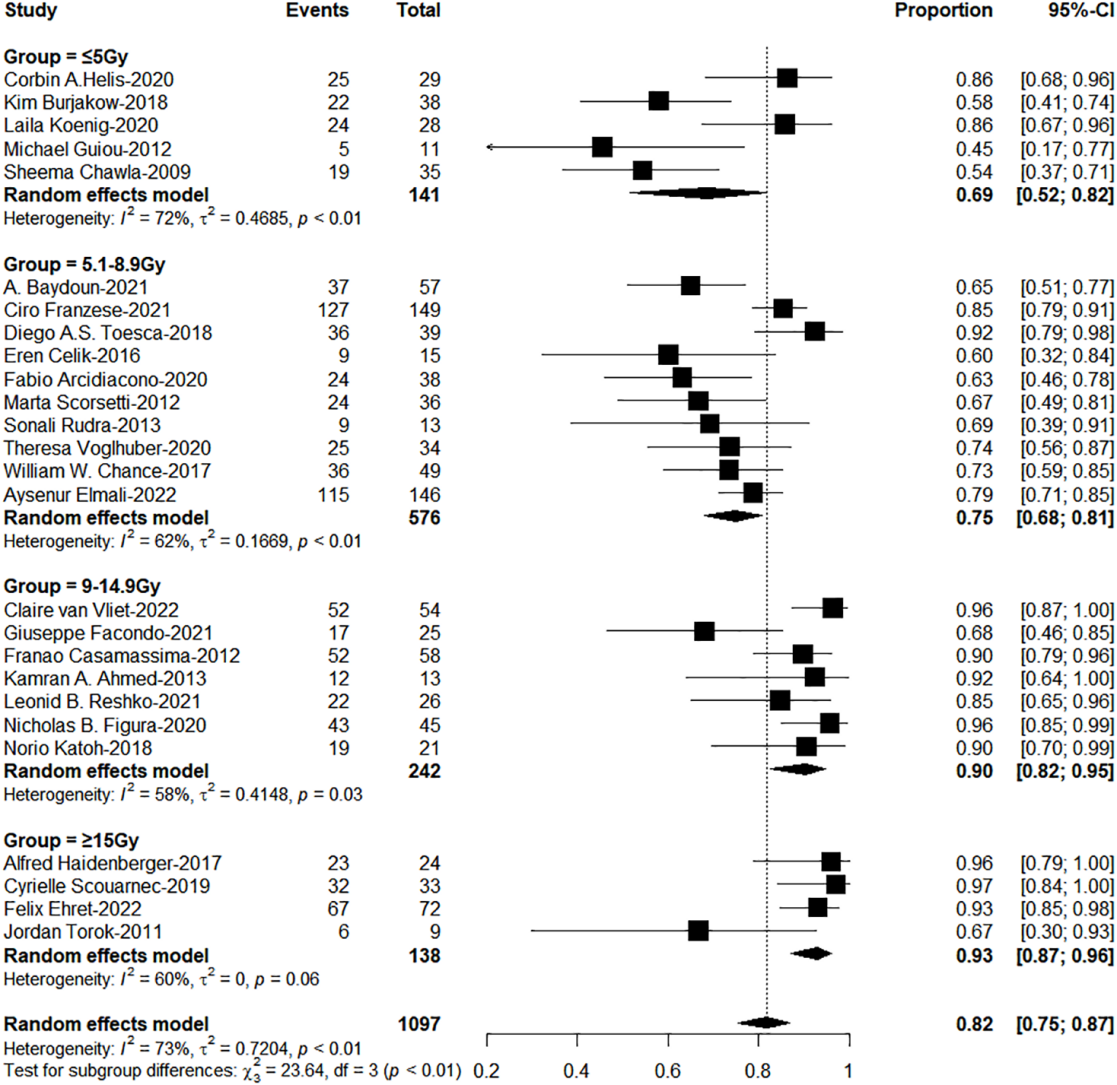
The forest plot of subgroup analysis for one-year local control by dose per fraction.

The subgroup analysis results showed the group of BED_10_ ≥ 100Gy have an excellent 1-year LC (<70Gy: 70-99Gy: ≥100Gy = 69%: 86%: 96%, χ*^2^ = *33.74, *P<* 0.0001, **Figure 2**). In clinical practice, due to the presence of surrounding critical organs, we often cannot escalate the radiation dose without the support of tracking technology. The majority of studies still have a total prescription dose ranging from 60 to 80 Gy. Therefore, we further performed subgroup analysis in the range of 60-80Gy (BED_10_) (N=21 studies, accounting for 64%) and the results showed 1-year LC in the group of dose per fraction≥9Gy (91%: 78%, χ*^2^ = *10.16, *P*=0.001, **Figure 3**). On the other hand, dose per fraction ≥ 9Gy have an excellent 1-year LC (≤5Gy: 5.1-8.9Gy: 9-14.9Gy: ≥ 15Gy = 68%: 75%: 90%: 94%, χ*^2^ = *27.93, *P =*0.0001, **Figure 4**). Even in the low tracking adoption group (0-20% patients, most of them without tracking adoption (15/18)), dose per fraction still significantly affected 1-year LC (N=17 studies, Qm =13.93, *P*<0.001), which rules out the role of tracking technology in local control rates for AM.

### The application of tracking significantly improved overall survival

As shown in **Table 1**, only tracking technology adoption has a strong correlation with 1-year OS (Qm=5.73; *P* = 0.017). SCLC, dose per fraction, tumor volume (PTV, GTV), tracking technology had strong effects on 2-year OS (Qm=10.04, 6.60, 4.87, 8.20, 8.75; *P* = 0.002, 0.010, 0.027, 0.004, 0.003). As shown in **Figure 2**, tracking technology adoption had a strong correlation with dose per fraction (The coefficient =0.61). But in the low tracking adoption group (0-20% patients, most of them without tracking adoption(15/18)), dose per fraction didn’t significantly affect 2-year OS (N =17 studies, Qm = 0.30, *P* = 0.584). The high-tracking adoption group received a median BED_10_ of 75.5Gy (t= -0.837, P =0.410), while the low-tracking adoption group received a median BED_10_ of 69.4Gy. These results suggested that, with regard to overall survival, the impact of fractionation doses may be contingent upon the use of tracking technology rather than the tracking technology upon fractionation doses.

**Figure 5:**
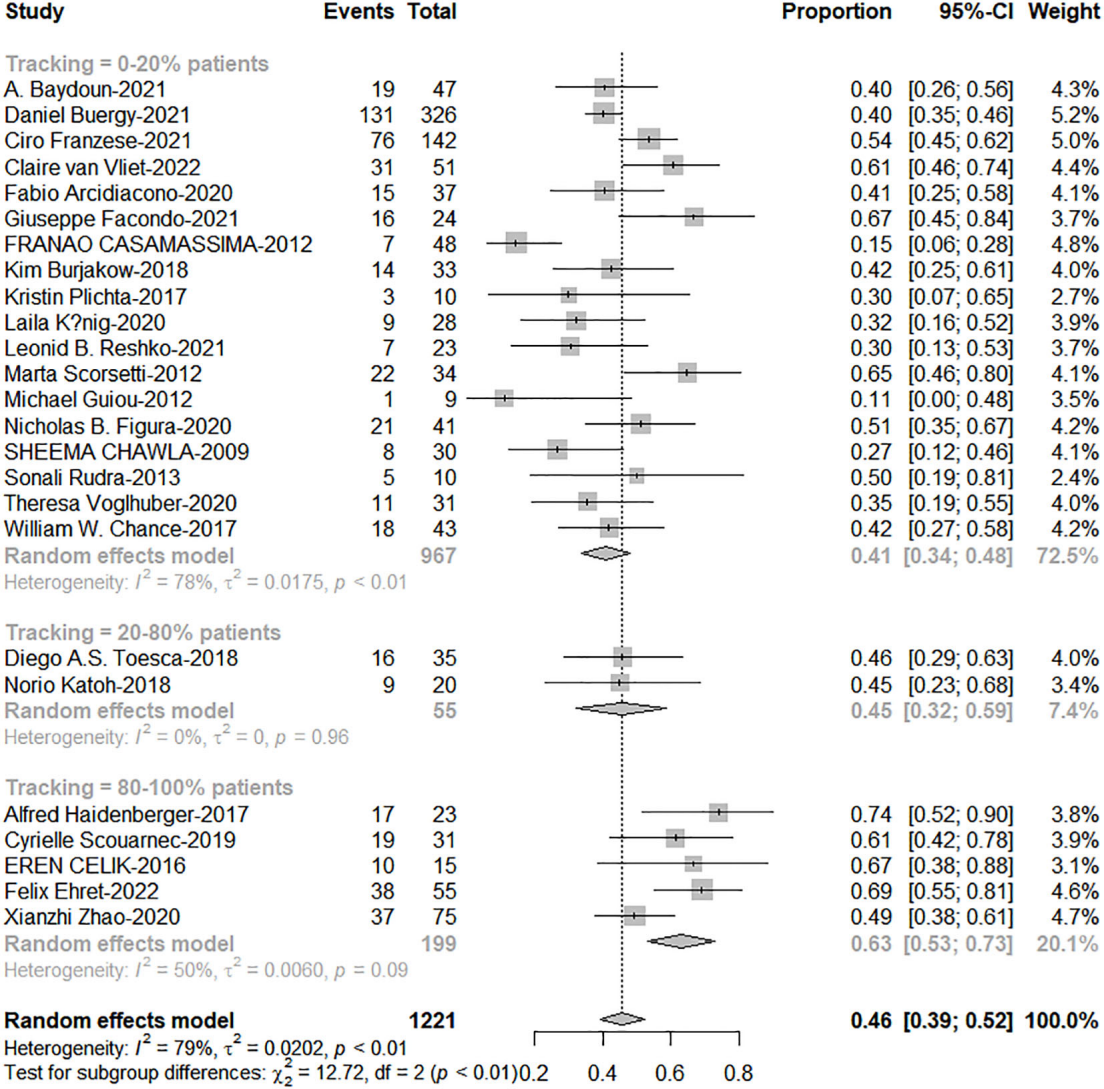
The forest plot of subgroup analysis for 1-year overall survival by tracking technology adoption rate.

**Figure 6:**
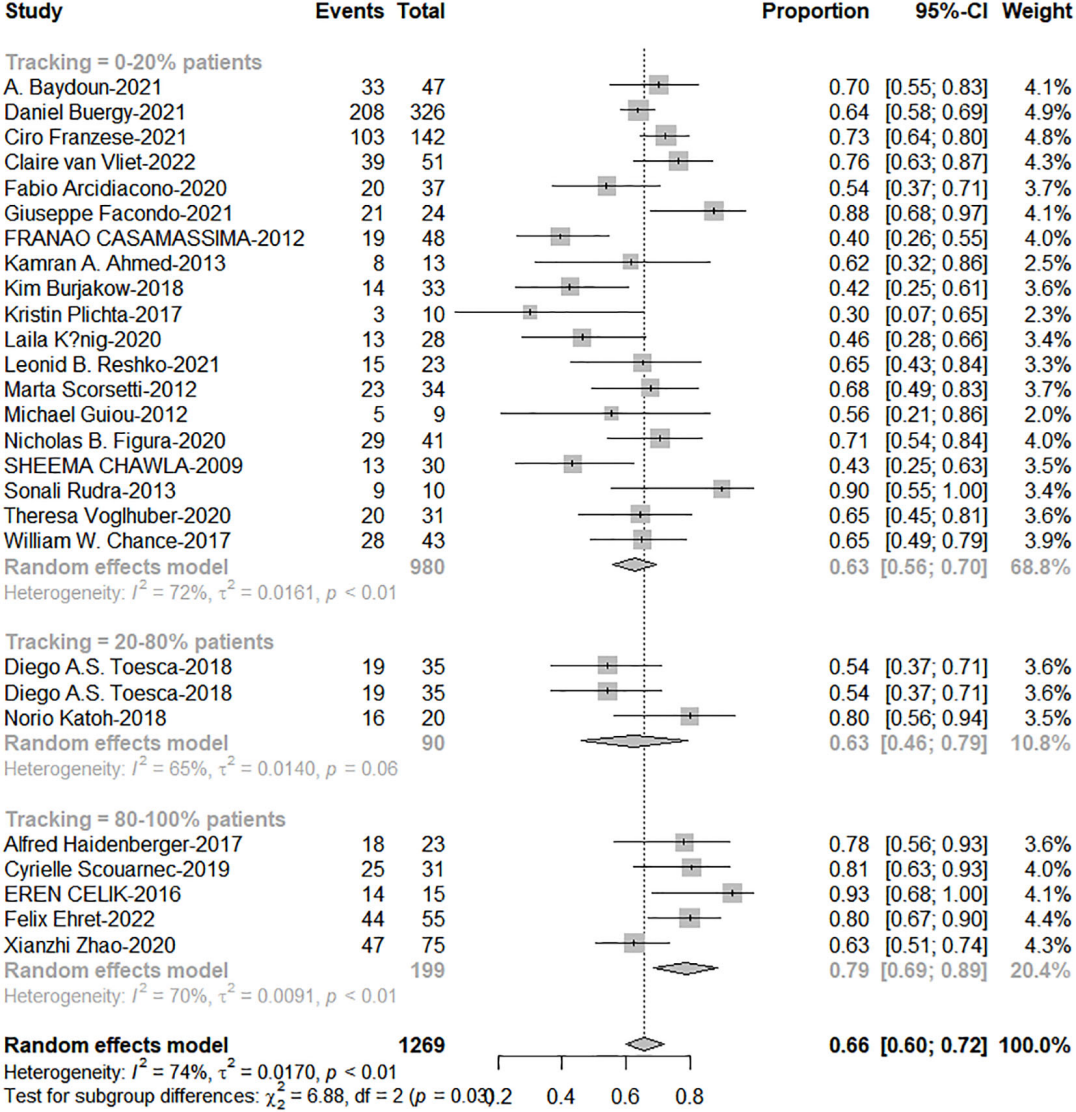
The forest plot of subgroup analysis for 2-year overall survival by tracking technology adoption rate.

The total proportion of tracking technology is only 19.3% (257/1332) from published articles. High tracking adoption group got excellent 1-and 2-year OS (0-20% patients: 20%-80% patients: 80%-100% patients = 63%: 67%: 79%, c2 = 6.88; P=0.038; 41%: 46%: 63%, c2 = 12.72, P=0.002, **Figures 5**, **6**). The side effects caused by the use of tracking technology accounted for 1.2% (3/257) of cases, all of which were associated with fiducial placement, including 1 case of spontaneous hematoma absorption, 1 case of pneumothorax, and 1 case requiring chest tube insertion, and these were concentrated in a single study (14). In high-tracking adoption group, the Grade 3+ toxicity response was 0.9% (2/213) and low-tracking adoption group, it was 1.1% (12/1091)(Differences were not statistically significant). Adrenal insufficiency morbidity was 1.8% (4/213) and 0.6% (6/979) separately because in high-tracking adoption group, three patients had only one remaining adrenal gland received SBRT.

## Discussion

Delivery of high-dose on adrenal gland can be challenging due to the proximity of risk organs such as the duodenum, small intestine, and stomach, with dose limit violations potentially leading to life-threatening complications (40). In clinical practice, compromises on target doses are necessary (4, 8). Moreover, the overuse of radiation can trigger the expression of certain proteins, such as Transforming Growth Factor (TGF), Indoleamine-pyrrole 2,3-dioxygenase (IDO), and Programmed cell Death-Ligand 1 (PD-L1), which contribute to an increase in immune-suppressive cells like Tregs and tumor-associated macrophages (TAMs) (41), and have harmful effects on the immune system’s ability to fight cancer cells. Thus, seeking a dose scheme under 100Gy of BED_10_ not only helps to protect the surrounding organs at risk, but also helps to weaken the local immunosuppression around the tumor.

Our study underscores the importance of fractionated doses and emphasizes that fractionated doses should not be less than 9 Gy. When it is not feasible to achieve the currently recommended BED10 ≥ 100 Gy total dose, our study offers a solution by increasing the fractionated dose. This finding aligns with experimental data (42–44), as doses greater than 8-10 Gy, as opposed to doses greater than 5 Gy, can result in rapid endothelial cell apoptosis, leading to extensive tumor cell death and severe hypoxia in the microenvironment. Furthermore, the release of tumor antigens during the death or dying process of tumor cells post high fractionated radiation can trigger an anti-tumor immune response within weeks to months, contributing to the formation of a local immune environment (45, 46). There is evidence that in animal experiments, a dose of 8-10 Gy is optimal, as fractionated radiation at 8 Gy, combined with CTLA-4 inhibitors, can induce a distant effect (47).

Our research demonstrates that the utilization of tracking technology not only contributes to extending the survival of patients with AM but is also highly safe. Real-time monitoring and tracking of tumor location is an emerging technology to enhance the precision of tumor radiotherapy (48). This technology allows for high-dose, high-accuracy irradiation of the tumor, improving treatment effectiveness while avoiding “off-target” effects in the target area, and reducing adverse effects on surrounding tissues (48, 49). A study published in the journal Nature Cancer suggests that damage to tissues caused by “off-target” radiotherapy can locally activate neutrophils in the normal repair process and Notch signal transduction, which can promote cancer metastasis and reduce overall survival (50). For under-irradiated tumors cells, insufficient radiation fails to kill them; instead, it enhances its malignancy and promotes invasion and metastasis (51, 52).

Therefore, the application of real-time tracking technology is essential, especially for patients with AM. We found that 91% of AM patients fall within the current definition of oligometastasis. Multiple studies have shown that aggressive treatment in oligometastatic patients can significantly extend survival (53–55). However, regrettably, the use rate of tracking technology is only 19% (257/1332) and the importance of tracking technology urgently needs to be widely promoted. Of course, we also need to be vigilant about selection bias. Because this portion of patients in the high fiducial adoption group may also have better medical support, which could potentially influence the overall survival outcome. Nevertheless, we cannot deny the importance of adopting tracking radiation therapy, and we hope that prospective studies in the future will confirm our findings.

We are the first to propose a study on the focus of fractional dose and tracking technology in the treatment of AM using SBRT. William C. Chen et al (39) collected 39 studies between 2009 and 2019 to perform first meta-analyze on AM by SBRT. They only analyzed the relationship between the total dose (BED10) and local control, as well as overall survival, and reached the conclusion that dose escalation contributes to local control. They did not analyze and find the significance of fractionation dose and tracking techniques, perhaps due to the inclusion of a large number of abstracts with insufficient information in their study. To extract more data and ensure data accuracy, we excluded all studies that had only abstracts and lacked full texts. Furthermore, due to the recent advancements in tracking technology and the development of imaging techniques, many new articles (14/out of 33) on SBRT treatment for AM have emerged. Therefore, there is an urgent need for updated meta-analyses in this regard.

Our study has some limitations that need to be acknowledged. The inconsistencies in radiation techniques and dosimetry, and the inherent biological inaccuracies in calculating BED by linear-quadratic (LQ) formulas, mean that the reported doses are only informative and should be carefully considered. As our study is a retrospective analysis, it cannot establish causal relationships, and more prospective studies are needed to verify our findings. The results of meta model-based estimates should be interpreted with caution, given the heterogeneity of tumor control estimates extracted from the literature and the variability of diametric data reporting, as well as the definitions and statistical methods used to report tumor control. Despite our efforts to collect data of the same quality, there were still some differences in detail.

## Conclusions

SBRT is a safe technique. Constrained by organs at risk, the clinical dose for treating AM often falls within the range of 40-80 Gy, especially in centers with low tracking adoption rates. But we recommend that the minimum dose per fraction should be set around 9 Gy to ensure treatment efficacy.Additionally, the use of tracking techniques may improve the survival rates of advanced AM patients and is strongly recommended. Prospective studies are needed to validate these discoveries.

## Data availability statement

The original contributions presented in the study are included in the article/**Supplementary Material**. Further inquiries can be directed to the corresponding author.

## Author contributions

XL had full access to all of the data in the study and takes responsibility for the integrity of the data and the accuracy of the data analysis. Concept and design: KK. Acquisition, analysis, or interpretation of data: all authors. Drafting of the manuscript: all authors. Critical revision of the manuscript for important intellectual content: KK and RK. Statistical analysis: XL. Obtained funding: CP. Administrative, technical, or material support: KK, CP. Supervision: KK, CP, RK. All authors contributed to the article and approved the submitted version.
