# Supplementary material for: Risk factors of local control in adrenal metastases treated by stereotactic body radiation therapy - a systematic review and meta-analysis
Source: Front Oncol. 2023 Nov 17;13:1193574. doi: 10.3389/fonc.2023.1193574 (PMC10691549; doi:10.3389/fonc.2023.1193574)

**Supplemental Figures**

Fig. A Flow diagram summarizing the article selection process.

33 studies included in meta-analysis

131 studies screened based on full text

913 records identified

Embase 633

Pubmed 136

Web of science 144

915 records identified:

Embase 633;

PubMed 137; Web of science 145

616 studies screened based title/abstract

98 studies excluded:

review/case report/erratum n=19

no only adrenal metastasis/SBRT n=5

technical data only n=14

redundant data n=8

no biological effective dose n=11

duplicate data n=8

no toxicity or outcome data n=19

no English article n=2

only abstract n=11

meta-analysis n=1

485 non-relevant records excluded

299 duplicate excluded

Fig. B-1 The forest plot of pooled 1-year local control (27 studies with 1463 lesions).


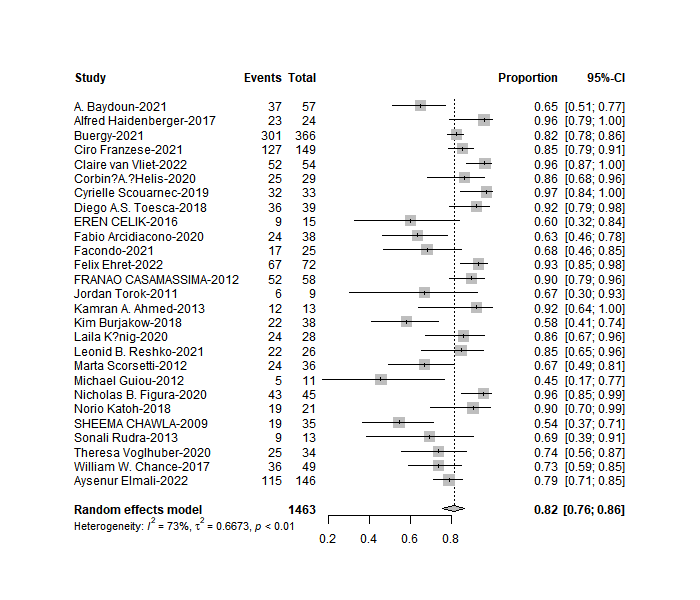


Fig. B-2 The forest plot of pooled 2-year local control (24 studies with 1351 lesions).


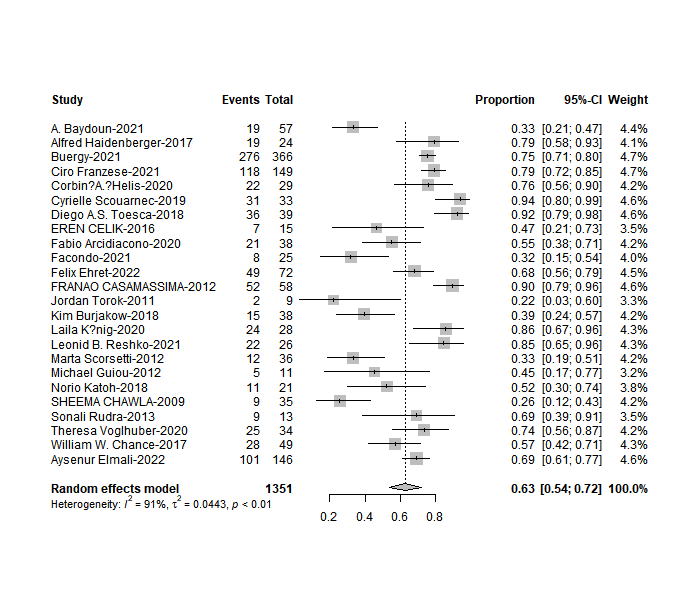


Fig.B-3 The forest plot of pooled 1-year overall survival (28 studies with 1385 patients).


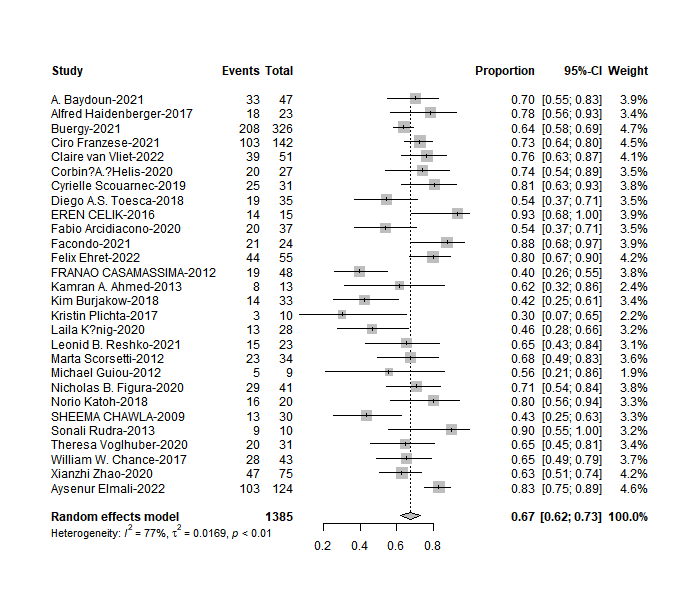


Fig.B-4 The forest plot of pooled 2-year overall survival (27 studies with 1372 patients).


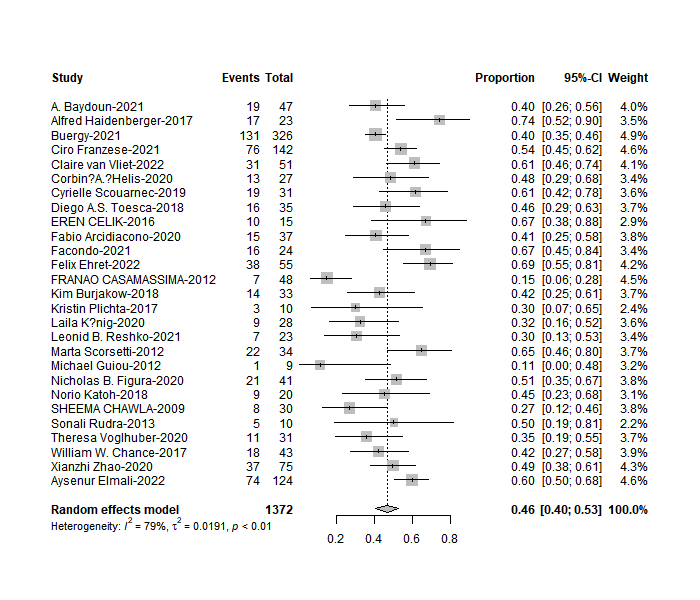


Fig.C-1 The funnel plot of 1-year local control (27 studies with 1463 lesions).


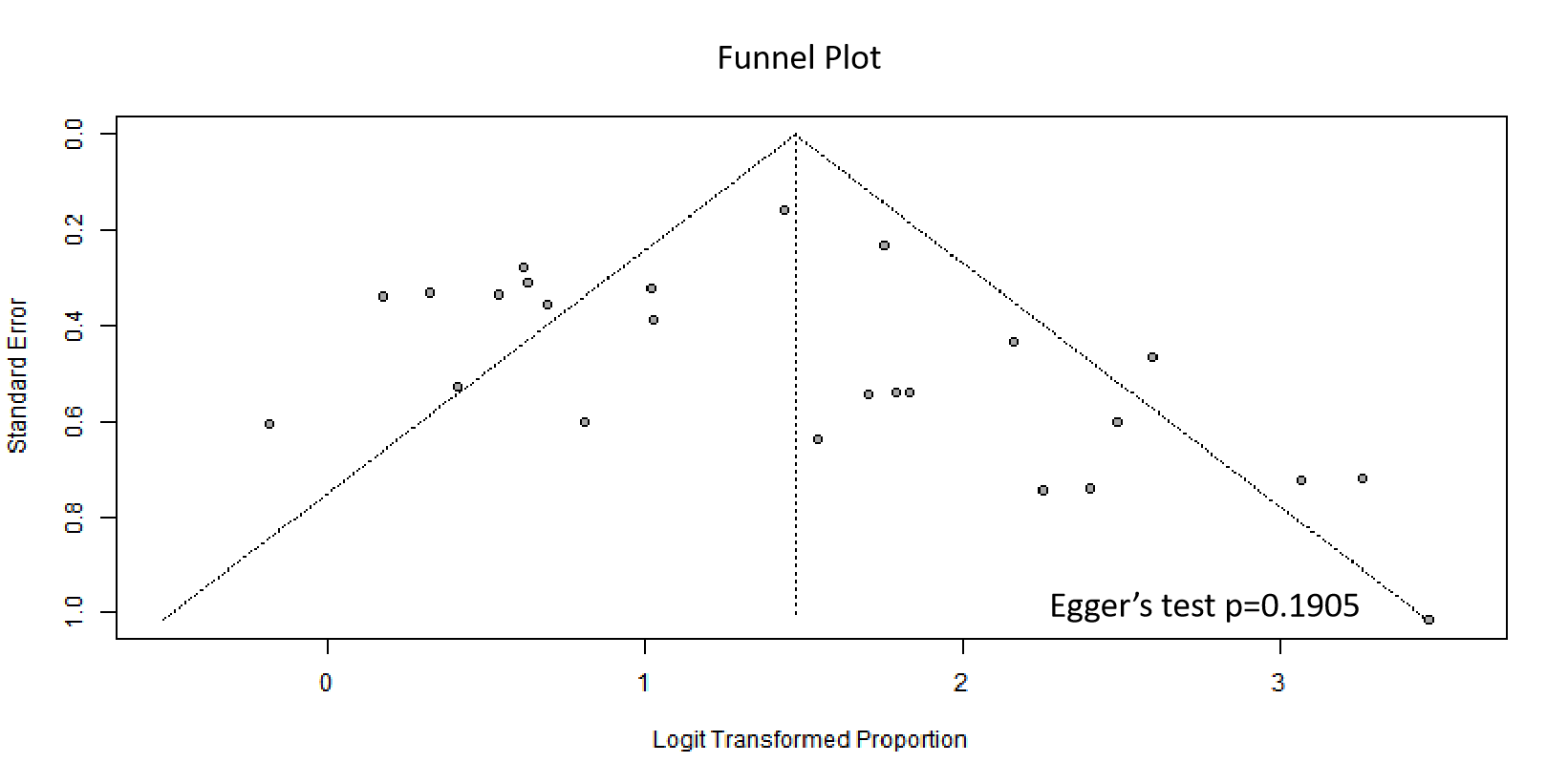


Fig. C-2 The funnel plot of pooled 2-year local control (24 studies with 1351 lesions).


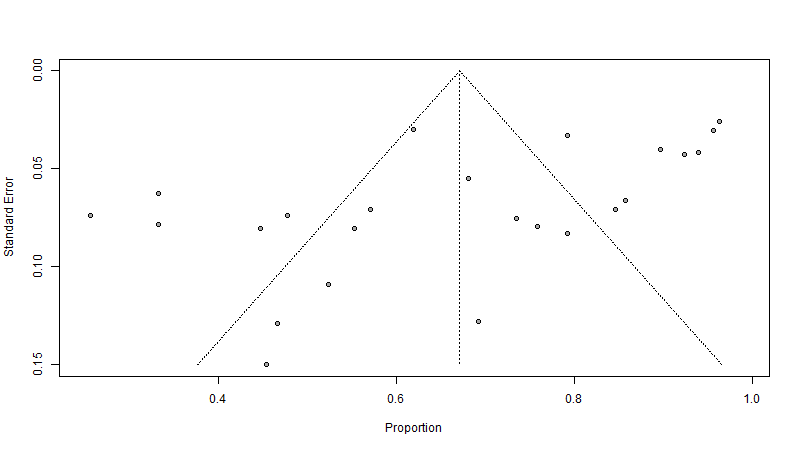


Fig.C-3 The funnel plot of pooled 1-year overall survival (28 studies with 1385 patients).


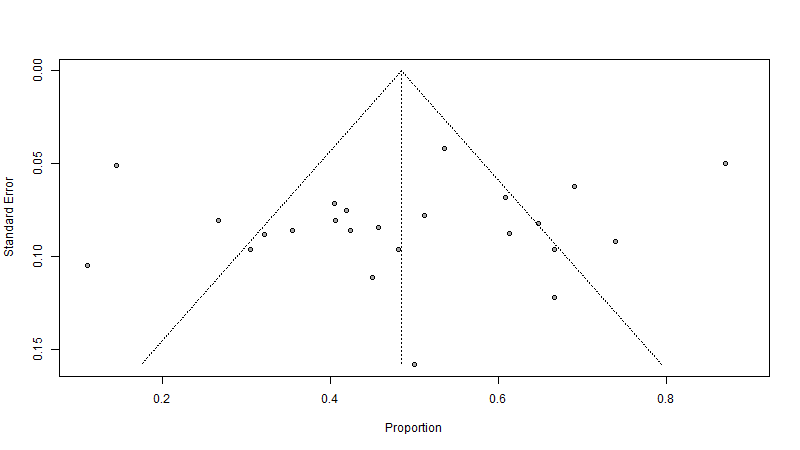


Fig.C-4 The funnel plot of pooled 2-year overall survival (27 studies with 1372 patients).


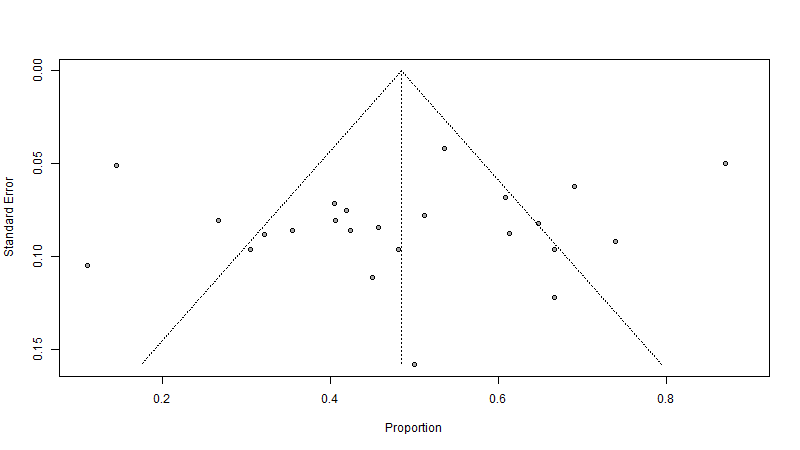


Fig. D-1 The sensitivity analysis of 1-year local control (27 studies with 1463 lesions).


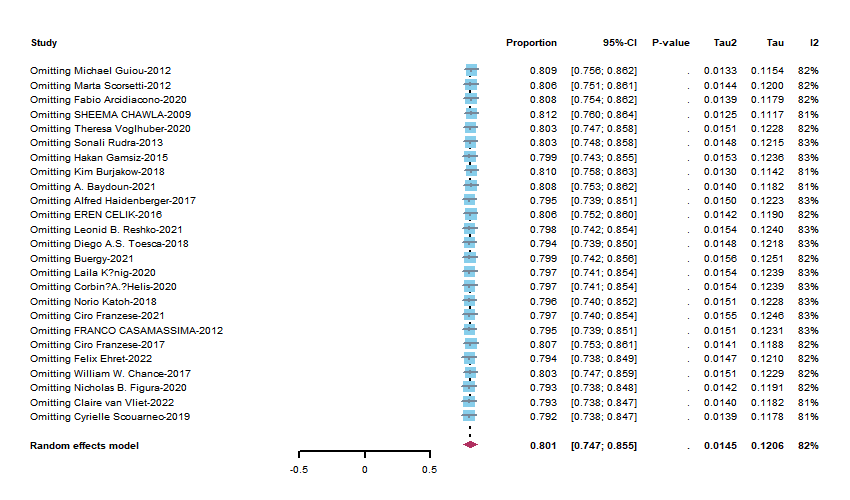


Fig. D-2 The sensitivity analysis of pooled 2-year local control (24 studies with 1351 lesions).


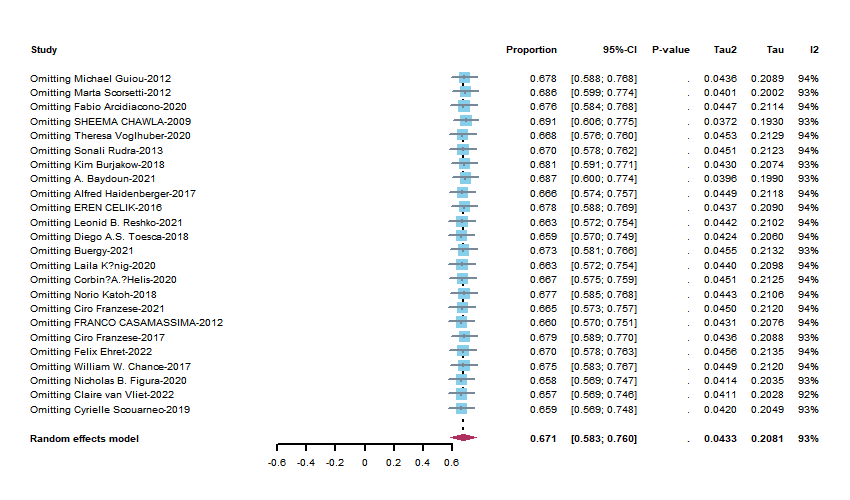


Fig.D-3 The sensitivity analysis of pooled 1-year overall survival (28 studies with 1385 patients).


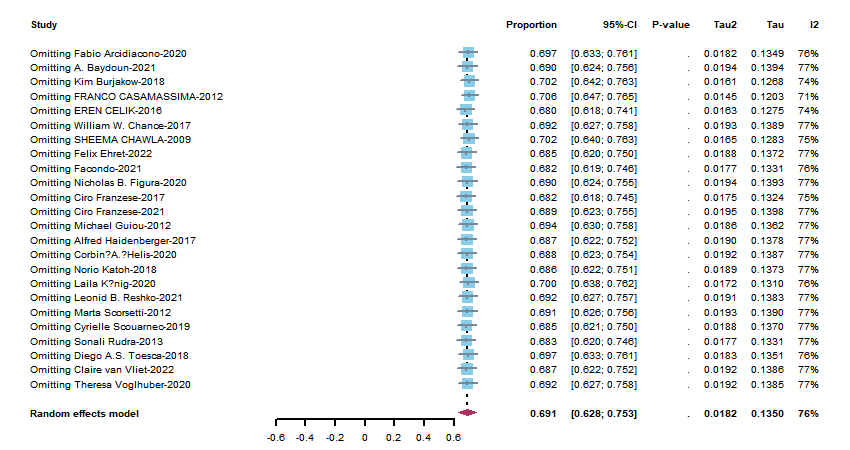


Fig.D-4 The sensitivity analysis of pooled 2-year overall survival (27 studies with 1372 patients).


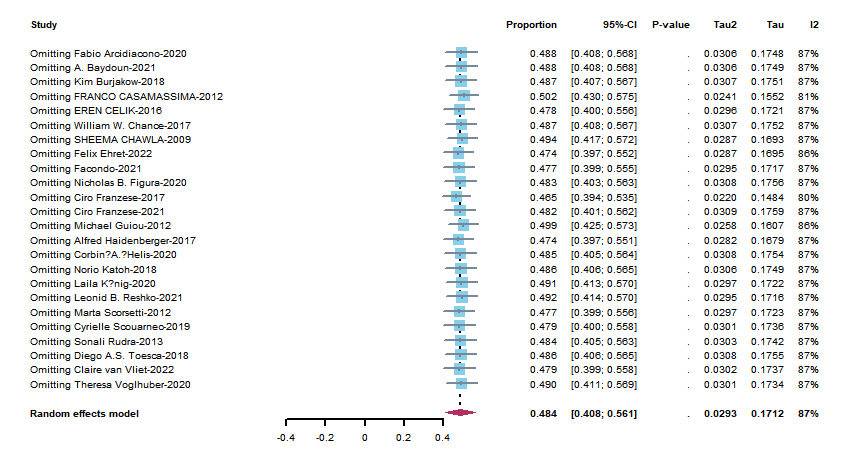

Supplement: Supplementary file 1 [file DataSheet_1.docx]
